# Risk Score for Predicting In-Hospital Mortality in COVID-19 (RIM Score)

**DOI:** 10.3390/diagnostics11040596

**Published:** 2021-03-26

**Authors:** Alejandro López-Escobar, Rodrigo Madurga, José María Castellano, Sara Velázquez, Rafael Suárez del Villar, Justo Menéndez, Alejandro Peixoto, Sara Jimeno, Paula Sol Ventura, Santiago Ruiz de Aguiar

**Affiliations:** 1 Pediatrics Department, HM Hospitales, Fundación de Investigación HM Hospitales, Facultad de Medicina, Universidad CEU San Pablo, 28015 Madrid, Spain; 2 Fundación de Investigación HM Hospitales, Faculty of Experimental Sciences, Universidad Francisco de Vitoria, 28015 Madrid, Spain; rodrigo.madurga@ufv.es; 3 Cardiology Department, Hospital Universitario HM Montepríncipe, Grupo HM Hospitales, Fundación de Investigación HM Hospitales, Facultad de Medicina, Universidad CEU San Pablo, Centro Nacional de Investigaciones Cardiovasculares, Instituto de Salud Carlos III, 28015 Madrid, Spain; jmcastellano@fundacionhm.com; 4 Anaesthesiology Department, Hospital Universitario HM Sanchinarro, Hospital Universitario Santa Cristina, Fundación de Investigación HM Hospitales, 28015 Madrid, Spain; sara.velazquezdiaz@usp.ceu.es; 5 Internal Medicine Department, Hospital Universitario HM Sanchinarro, Fundación de Investigación HM Hospitales, 28015 Madrid, Spain; rsuarezdelvillar@hmhospitales.com; 6 Emergency Department, Grupo HM Hospitales, Fundación de Investigación HM Hospitales, Facultad de Medicina, Universidad CEU San Pablo, 28015 Madrid, Spain; jmenendez@hmhospitales.com; 7 Facultad de Medicina, Universidad CEU San Pablo, 28015 Madrid, Spain; alejandro.peixoto@icloud.com; 8 Pediatrics Department, Hospital Universitario HM Puerta del Sur, Móstoles, Fundación de Investigación HM Hospitales, Facultad de Medicina, Universidad CEU San Pablo, 28015 Madrid, Spain; sarajimeno@hotmail.com; 9 Pediatrics Department Hospital Universitario HM Nens, Fundación de Investigación HM Hospitales, 08009 Barcelona, Spain; paulasolventura@hotmail.com; 10 Medical Management, Hospital Universitario HM Puerta del Sur, Móstoles, Fundación de Investigación HM Hospitales, 28015 Madrid, Spain; sruizdeaguiar@hmhospitales.com

**Keywords:** COVID-19, neutrophil-to-platelet ratio, NPR, neutrophil-to-lymphocyte ratio, NLR, hemogram-derived-ratios

## Abstract

Infection by SARS-CoV2 has devastating consequences on health care systems. It is a global health priority to identify patients at risk of fatal outcomes. 1955 patients admitted to HM-Hospitales from 1 March to 10 June 2020 due to COVID-19, were were divided into two groups, 1310 belonged to the training cohort and 645 to validation cohort. Four different models were generated to predict in-hospital mortality. Following variables were included: age, sex, oxygen saturation, level of C-reactive-protein, neutrophil-to-platelet-ratio (NPR), neutrophil-to-lymphocyte-ratio (NLR) and the rate of changes of both hemogram ratios (VNLR and VNPR) during the first week after admission. The accuracy of the models in predicting in-hospital mortality were evaluated using the area under the receiver-operator-characteristic curve (AUC). AUC for models including NLR and NPR performed similarly in both cohorts: NLR 0.873 (95% CI: 0.849–0.898), NPR 0.875 (95% CI: 0.851–0.899) in training cohort and NLR 0.856 (95% CI: 0.818–0.895), NPR 0.863 (95% CI: 0.826–0.901) in validation cohort. AUC was 0.885 (95% CI: 0.885–0.919) for VNLR and 0.891 (95% CI: 0.861–0.922) for VNPR in the validation cohort. According to our results, models are useful in predicting in-hospital mortality risk due to COVID-19. The RIM Score proposed is a simple, widely available tool that can help identify patients at risk of fatal outcomes.

## 1. Introduction

The coronavirus disease 2019 (COVID-19) has become a pandemic since the outbreak in Wuhan City, China, in December 2019, affecting over one hundred million patients worldwide by January 2021, with a death toll over two million [1]. Due to the high number of cases, many health systems have collapsed due to the fast evolution from a banal disease, which can be treated on an outpatient basis, to a disease with systemic complications including severe acute respiratory failure requiring Intensive Care Unit (ICU) admission, and death [2].

Generating an accurate prediction tool to predict clinical course of the disease could prove very helpful in risk stratification, clinical decision-making, rational resource optimization, including administration of drugs in order to avoid serious adverse effects and ultimately reduce the lethality. Several studies have proposed simple clinical scores to identify patients at risk of progression to the more severe forms of COVID-19, which have included older age, dyspnea, presence of comorbidities, higher levels of LDH, C-reactive protein (CRP), and direct bilirubin have been also associated with poor outcomes [3,4,5,6,7]. Severe COVID-19 has been correlated with increased levels of circulating interleukins and other inflammatory biomarkers, resulting in the so-called "cytokine storm". The implementation of these markers in scores could help fine tune the accuracy of a clinical score in the early detection of severe cases of COVID-19.

Similar to other viral infections, COVID-19 patients display lymphopenia and thrombocytopenia. Recent data has provided observational proof linking the hemogram-derived ratios Neutrophil-to-Lymphocyte ratio (NLR) and Platelet-to-lymphocyte ratio (PLR) to the more severe cases of COVID-19 [6,7,8,9]. Our group has reported the usefulness of these ratios in COVID-19 including the novel neutrophil-to-platelet ratio (NPR), which is the ratio between the count of neutrophils and the count of platelets [9,10,11]. Following alveolar viral damage by SARS-CoV-2 a hyperinflammatory response has been identified in moderate to severe cases and endothelial cells may be playing an important role as a driver of inflammation mediating the release of cytokines. In this context, activated platelets and neutrophils play a determining role in microvascular occlusion during the thromboinflammatory phase of the disease so NPR may be useful based on the biological plausibility of higher total neutrophils count and lower total platelets count observed among the most severe COVID-19 cases compared to more mild ones [10,11]. 

We propose these ratios should be incorporated with other epidemiological and clinical variables in a nomogram to help identify moderate to severe cases of COVID-19, given their capacity to signal a combination of hyperinflammatory response and microvascular occlusion that has been identified in the pathophysiology of moderate to severe cases [12,13]. Early detection of patients affected by COVID-19 who will have worse evolution is a priority. Based on this proposal we aimed to develop and validate a risk score for predicting in-hospital mortality in COVID-19 (Risk of In-hospital Mortality Score in COVID 19, RIM Score).

## 2. Methods

COVID-19 patients that were hospitalized at any of the 10 hospitals of the HM Hospitales Group across different regions in Spain (including Madrid, Barcelona, and Galicia, Spain) from 1 March to 10 June 2020, were retrospectively included in the study. Clinical and laboratory data measurements were available up to and including 24 June 2020.

During the study period, due to the dramatic pandemic situation with a multitude of admitted patients and a shortage of PCR tests, there were changes in the diagnostic protocol proposed by the Spanish Ministry of Health. For several weeks, the diagnosis of COVID-19 was based solely on clinical characteristics and radiological criteria.

The study was conducted according to basic ethical principles and the development followed the standards of Good Clinical Practice and the principles enunciated in the latest Declaration of Helsinki (2013) and the Oviedo Convention (1997). The study protocol was approved by the ethics committee of HM Hospitales (approval number 20.03.1573-GHM). Informed consent was obtained from all subjects as they all agreed to access and analyze their medical history data.

**Scheme 1:**
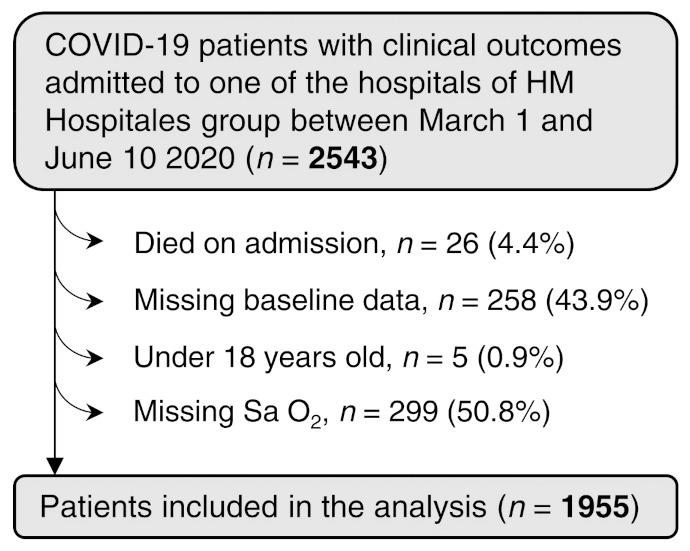
Patients Flowchart.

A total of 2543 COVID-19 patients were admitted during the study period. Patients having missing SaO_2_ at admission (*n* = 299) or laboratory data in the first 24 hours of admission (*n* = 258), whose age was under 18 years old (*n* = 5) or died at hospital admission (*n* = 26), were excluded from the analysis. From the total of 2543 patients admitted, 1955 (76.8%) were included in the final analysis as shown in the flow diagram in Scheme 1.

## 3. Predictive Variables

We selected a set of variables, as measured at hospital entry, which were found to be predictors of COVID-19 mortality, the outcome of this research, in previous studies [9,10,11]. Those variables included age, sex, oxygen saturation <90%, level of CRP, NPR and NLR. NLR is the ratio between the count of neutrophils (×10^9^ cells/L) and the count of lymphocytes (×10^9^ cells/L) and NPR is the ratio between the count of neutrophils (×10^9^ cells/L) and the count of platelets (×10^11^ cells/L). In addition, the rates of change of both proportions of blood cell counts in the first week of hospital admission (Velocity of NPR (VNPR) and Velocity of NLR (VNLR), respectively) were collected. VNPR and VNLR were measured as the percent of change from the initial measurement per day.

Because NPR and NLR are highly correlated [9,10,11] we constructed four different models. On one hand, we built two models with those variables that were measured at hospital admission, including NLR on the first one and NPR on the second one. On the other hand, we built two additional models based on the scores of the two previously described models and the respective hemogram ratio rate of change, VNLR and VNPR.

The latter therefore include time dependent variables in addition to the initial risk score.

## 4. Score Construction and Validation

Imputation for missing data was considered for those variables with less than 2% of missing values, considered as missing as random (MAR). All missing values were from the numeric variable CRP and were imputed using predictive mean matching with the R package “mice” [14]. Diagnosis plots from imputation are shown in Figure S1. 

The 1955 patients included in the study were randomly divided in training cohort (*n* = 1310, 2/3) and validation cohort (*n* = 645, 1/3). Variables were summarized as median (IQR) for numeric variables and by number (%) for categorical variables. Comparison between the two cohorts was conducted using Mann-Whitney U test (two tailed), Chi-square test or Fisher test when required. 

NLR, NPR and CRP values were classified as low and high using the third quartile of survivors in the training set as a threshold. These thresholds were selected because it was observed that for all three variables the median of non-survivors was higher than the third quartile of survivors. VNLR and VNPR were categorized as positive (>0) or non-positive otherwise.

We used the training cohort to train the logistic regression models, augmented with 10-fold cross validation for internal validation. Those variables that were consistently statistically significant were used to construct the risk scores. The R package “rms” was used to generate the calibration curves. The calibration curve reflects the relation between the predicted probability (abscissa) and the actual probability (ordinate), measured as the incident rate. The accuracy of the risk scores was assessed and compared using the area under the receiver-operator characteristic curve (AUC). ROC curves were generated using the R package “pROC” [15]. To evaluate the clinical application of the models we used the decision curve, which were generated using the R package “rmda”. Calibration curves, ROC curves and decision curves were generated using 1000 bootstrap resamples.

Sensitivity analysis of the four models were performed by removing those patients without PCR test and repeating the model construction on the PCR tested patients.

Statistical analysis was performed with R software (version 4.02). We considered statistically significant those comparisons with *p*-value < 0.05.

Reporting of the study conforms to broad TRIPOD reporting guidelines [16].

## 5. Results

### 5.1. Training and Validation Cohort Characteristics

Clinical, epidemiological, and laboratory data for 1955 patients admitted to HM Hospitales Group due to COVID-19 infection from 1 March to 10 June 2020, were included for analysis. The median age of patients was 69 (57–80) and 60.1% were men (Table 1). All patients were initially assessed in the Emergency Department where complete blood work was carried out. Infection by SARS-CoV-2 was confirmed in 1827 (93.6%) patients. The remaining 128 patients presented clinical and/or radiological signs compatible with COVID-19, as per protocol. 

Based on previous reports [9,10,11], we selected age, sex, oxygen saturation <90%, level of CRP, NPR and NLR at hospital admission and VNPR and VNLR for analysis and model development. Characteristics and laboratory results are summarized in Table 1. Two hundred and ninety patients (14.8%) died and one hundred forty-six (7.5%) required admission at ICU. 

One thousand three hundred and ten patients (67%) were included in the training cohort and six hundred and forty-five (33%) in the validation cohort. No statically differences were found between both cohorts for any of the analyzed variables (Table 1). 

In the training cohort, at the time of hospital admission, baseline clinical differences were observed between patients who died and those who were discharged, including age (83 (75–89) vs. 66 (54–78, *p* < 0.0001)), sex (67% vs. 58.8% males, *p* = 0.037), SaO_2_ (SaO_2_ < 90% 51% vs. 18.5%, *p* < 0.0001) and level of CRP (114.94 (71.17–218.78) vs. 53.8 (21.63–11.83)). Patients who died presented significantly higher baseline values of NLR (8.74 (4.65–14.96) vs. 3.96 (2.59–6.86), *p* < 0.0001)) and NPR (3.5 (2.41–4.93) vs. 2.18 (1.58–3.03), *p* < 0.0001)) and significantly higher rate of change in NLR/VNLR (0.0%/day (0.0–26.6) vs. 0.0%/day (−9.1–4.67), *p* < 0.0001)) and NPR/VNPR (0.0%/day (−1.7–8.8) vs. −3.8%/day (−9.7–0.0), *p* < 0.0001)) than those who were discharged (Table 2).

### 5.2. NLR and NPR Models

We developed two logistic regression models that integrate 5 variables at the patient hospital entry (Table 3).

**Figure 1:**
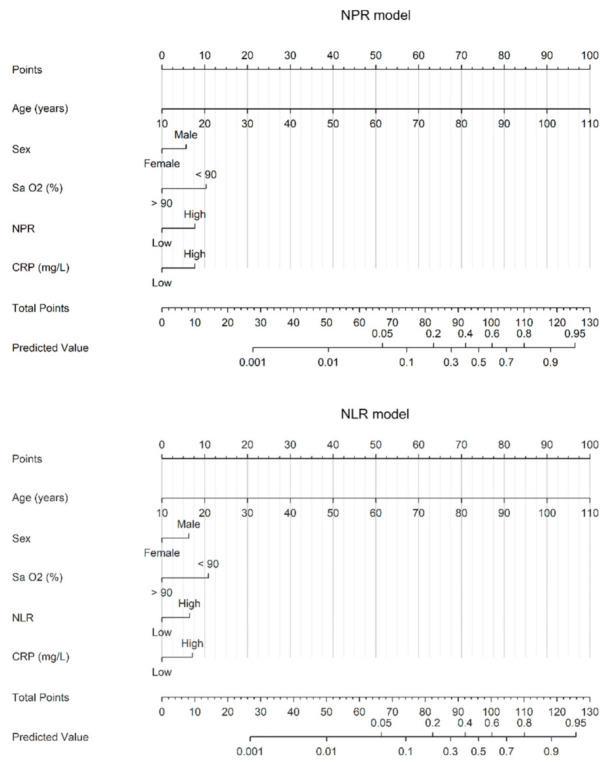
Nomograms of the NPR and NLR models. Abbreviations: SaO_2_, oxygen saturation; NLR (high: >6.9), neutrophil-lymphocyte ratio; NPR (high: >3.0), neutrophil-platelets ratio; CRP (high: >112 mg/L), C-reactive protein.

One of the models considers NLR while the other uses NPR. For both models the dependent variable was the patient status at the outcome. The respective predictive nomograms are shown in Figure 1. The continuous variable age was found to be almost linearly correlated with mortality (Figure S2A).

**Figure 2:**
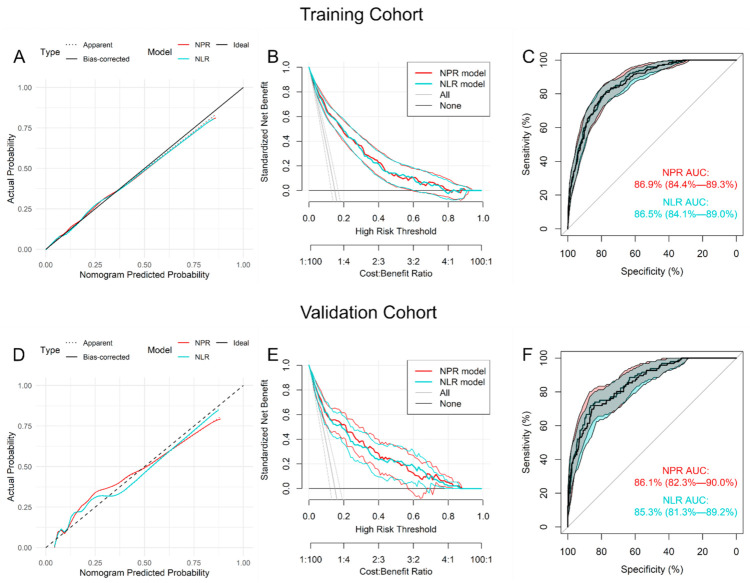
Evaluation of the prediction value of the nomograms NPR (red) and NLR (blue) in the training (**A–C**) and validation (**D–F**) cohorts. (**A**,**D**) Calibration plots (pointed lines represent the apparent calibration curves, solid lines represent the bias-corrected calibration curves obtained from the 1000 bootstrap and dashed line represent the ideal calibration curve), (**B**,**E**) decision curves and (**C**,**F**) ROC curves of the nomogram score. Abbreviations: NLR, neutrophil-lymphocyte ratio; NPR, neutrophil-platelets ratio.

The calibration curve for exitus outcome probability showed a good agreement between the predicted and actual probabilities in both the training and the validation cohorts (Figure 2A,D). Additionally, decision curve analysis showed that the nomograms’ predicted probabilities had a superior net benefit for both NLR and NPR models, with none of them showing a better performance than the other (Figure 2B,E).

Both models obtained almost the same area under the ROC curves (AUC) with 0.865 (95% CI: 0.841–0.89) for the NLR model and 0.869 (95% CI: 0.844–0.893) for the NPR model in the training cohort (Figure 2C). When the nomogram was applied to the validation cohort, a slightly, but almost negligible, decrease in the AUC was observed for both models. NLR model obtained an AUC of 0.853 (95% CI: 0.813–0.892) while NPR model obtained an AUC of 0.861 (95% CI: 0.823–0.900) (Figure 2F). Sensitivity analysis was performed removing those patients that do not have been PCR tested. The OR for each variable remains invariant with a slightly decrease in CRP for the NPR model, while CRP and NLR became marginally significant for the NLR model (Figure S3A).

### 5.3. Addition of the Rates of Change to the Models

The models mentioned above provide a predicted probability of death from variables measured at hospital admission. It is important to update the predicted probability with the evolution of the patient. For that purpose, we developed two additional models that were built with the predicted probabilities obtained at hospital entry and with the evolution of either NLR or NPR, as appropriate, measured as the rate of change in percentage from the value at entry per day. Predicted probabilities for NLR and NPR were observed to be almost linearly related with mortality (Figure S2B).

**Figure 3:**
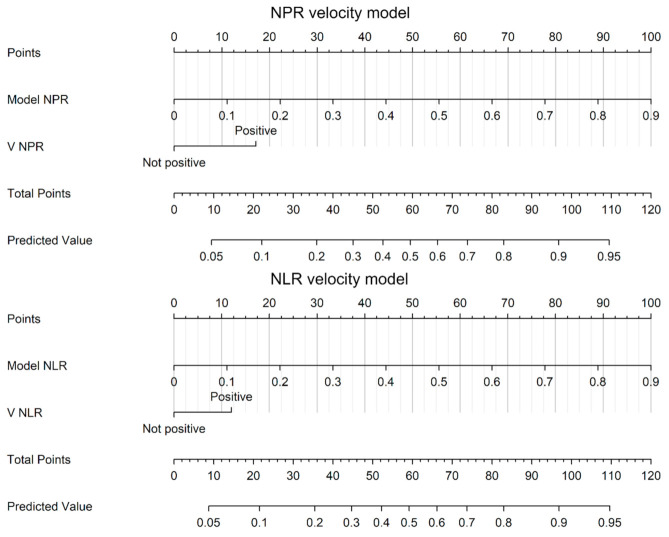
Nomograms of the NPR and NLR velocity models. Abbreviations: NLR, neutrophil-lymphocyte ratio; NPR, neutrophil-platelets ratio; VNLR, velocity neutrophil-lymphocyte ratio; VNPR; velocity neutrophil-platelets ratio; VNLR and VNPR categorized as positive (>0).

The nomograms constructed from the logistic regression models are shown in Figure 3. 

**Figure 4:**
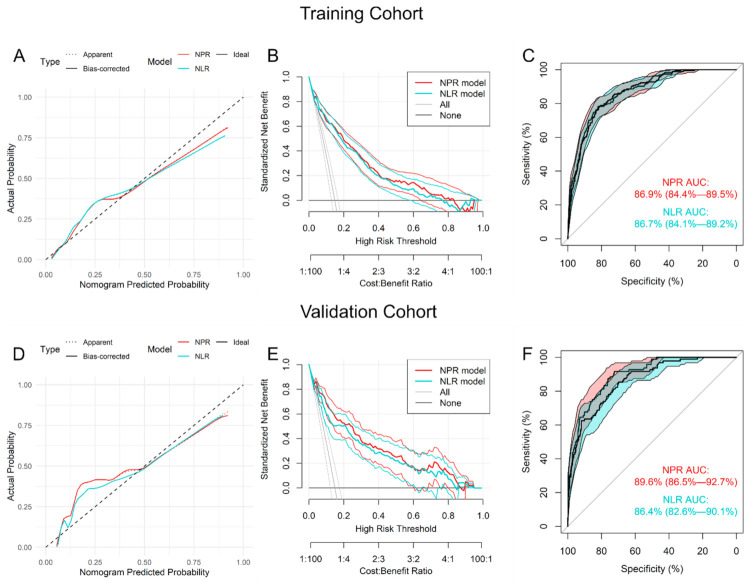
Evaluation of the prediction value of nomograms VNPR (red) and VNLR (blue) in the training (**A–C**) and validation (**D–F**) cohorts. (**A**,**D**) Calibration plots (pointed lines represent the apparent calibration curves, solid lines represent the bias-corrected calibration curves obtained from the 1000 bootstrap and dashed line represent the ideal calibration curve), (**B**,**E**) decision curves and (**C**,**F**) ROC curves of the nomogram score. Abbreviations: NLR, neutrophil-lymphocyte ratio; NPR, neutrophil-platelets ratio; VNLR, velocity neutrophil-lymphocyte ratio; VNPR; velocity neutrophil-platelets ratio.

The calibration curves showed a good agreement between predicted and actual probability of death in the validation cohort for both NLR-VNLR and NPR-VNPR models (Figure 4A). However, an undervaluation can be appreciated for predicted probabilities between 0.15 and 0.3 in the validation cohort (Figure 4D). Nevertheless, decision curve analysis showed a superior net benefit for the predicted probabilities obtained with NLR and NPR nomograms in the training and validation cohorts (Figure 4B,E).

The AUC after the incorporation of the NLR and NPR rate of change show no significant variation in comparison to the entry models, with an AUC of 0.867 (95% CI: 0.841–0.892) for NLR based model and 0.869 (95% CI: 0.844–0.895) for NPR based model (Figure 4C). A slight increase was observed in the AUC when tested in the validation cohort: AUC of NLR based model 0.864 (95% CI: 0.826–0.901), AUC of NPR model 0.896 (95% CI: 0.865–0.927) (Figure 4F). Sensitivity analysis showed a slightly but not significant decrease in the OR of the predicted probability of the entry models for both NLR and NPR (Figure S3B).

An interactive version of the RIM Score COVID is available at https://calculadoracovid.wordpress.com/.

## 6. Discussion

According to our results, RIM Score is an effective and easy tool for predicting risk of in-mortality in COVID-19 patients. We developed four models, two with NLR, a useful hemogram-derived ratio more widely reported for several studies, and two with the NPR, a novel hemogram-derived-ratio proposed by our group. According to our results, no significant differences were found between NLR and NPR models, however NPR models resulted more robust in the sensitivity analysis.

When incorporating the rate of change of the hemogram-derived ratios to the models, we appreciate that both, NLR and NPR models, tend to underestimate mortality for low predicted probabilities and slightly overestimate it at high predicted probabilities. Although VNLR seems to be more calibrated than VNPR, the ROC curve of VNPR showed a slightly better performance. Because the VNPR and VNLR models are built on the entry models of the respective hemogram-derives ratios and, as NPR showed to be more robust, we recommend using the NPR and VNPR models.

Several studies have published clinical scores trying to predict the patients affected by COVID-19 at risk of worse outcomes [3,4,5,6,7,17]. The scores and nomograms published to date are much more complex since they include many more parameters (some up to 23) and the predictability is lower than that reported by ours. Moreover, none of them combines the risk of mortality on admission with the predictability of risk of in-hospital death during admission for COVID-19 and most of these studies shown important methodological limitations including small patient samples, unrepresentative selection of the control patients, or short or incomplete follow-up [18].

In the current work, we present four scores, two of them are constructed with data from the first encounter with the patient at the Emergency Department and the other two models using data from the admission episode incorporating information from hemogram during hospitalization. The parameters used five at admission and one during evolution, are easily accessible, easily measured, routine, and affordable in any hospital environment. The predictive value of mortality of these nomograms renders them useful in clinical practice, showing an AUC of 0.861 and 0.853 in the validation cohorts for NPR and NLR models respectively, and an AUC of 0.896 and 0.864 in the validation cohorts for VNPR and VNLR models respectively. 

Early identification of patients at risk of moderate to severe forms of COVID-19 could condition a more energetic clinical behavior in the emergency room and lower admission thresholds, building upon the data that our group has provided demonstrating the usefulness of the hemogram-derived ratios in patients affected by COVID-19 with worse evolution, especially the novel NPR [9,10,11]. Adding the evolution of 3 parameters derived from a simple hemogram (neutrophils, lymphocytes and platelets) and including their rate of change with respect to the predicted value obtained in the nomogram could condition a different or more energetic therapeutic attitude if the hypothetical pro-inflammatory state worsens, and anti-inflammatory treatments would then be implemented in a timely manner.

Some factors such as age, hypoxemia, altered NLR and increased acute phase reactants have been identified as risk factors for mortality and worse prognosis. Our results are similar to those reported by other groups [9,10,11] and these findings would reflect an underlying inflammatory state that would become evident when weighted in the nomogram by combining the hemogram with the other identified factors. The use of hemogram-derived ratios, including the novelty NPR, have shown to be independent markers of mortality and worse prognosis in patients with COVID-19 [9,10,11].

Our study shows some limitations which should be addressed. Diagnostic protocols in the early phase of the pandemic changed due to shortage of PCR kits, as explained earlier. Although the study population only included patients within Spanish territory, given the diverse demographic variation which included patients from three regions of Spain, we expect the model perform similarly in other populations. This is a retrospective study and data were collected entirely from electronic reports; therefore, important information might be missed. We had to exclude some patients due to incomplete data at the Emergency Department. In the first nomogram we focused on the patients at hospital admission but in second nomogram we include a single parameter, the rate of change of NLR and NPR, which could be influenced by concomitant treatments and factors during hospitalization that might influence mortality such as corticosteroids. These treatments may have had an impact over blood cell counts and may be partly responsible for increased rates of change. However, various studies regarding the prognostic value of NLR in inflammatory diseases have shown a reduction in the ratio in patients under corticosteroid treatment [19,20].

## 7. Conclusions

We have developed RIM Score COVID, an easy and practical quantitative prediction tool which uses routine parameters used in nearly every health care setting where COVID-19 patients are being attended worldwide, at no extra cost or needing additional laboratory equipment. These assessments provide additional predictive value of mortality risk with a high value of accuracy. The parameters used in the nomogram are objective, easy to obtain, and reproducible in most health care centers. Further studies are needed to determine the real-world use of these nomograms in helping clinical judgement.

## Figures and Tables

**Table 1:** Baseline Demographics, clinical and laboratory characteristics. Median value (interquartile range).

	Total (*n* = 1955)	Training Cohort (*n* = 1310)	Validation Cohort (*n* = 645)	*p* Value
Age	69 (57–80)	70 (57–81)	68 (57–79)	0.13
Sex (Male)	1175 (60.1%)	786 (60%)	389 (60.3%)	0.93
SaO_2_ < 90%	441 (22.6%)	305 (23.3%)	136 (21.1%)	0.3
NLR	4.32 (2.71–7.97)	4.36 (2.73–8.05)	4.27 (2.65–7.83)	0.64
NPR	2.34 (1.65–3.35)	2.30 (1.65–3.34)	2.38 (1.66–3.38)	0.52
VNLR	0.00 (−8.88–6.36)	0.00 (−8.30–6.37)	0.00 (−9.67–6.33)	0.094
VNPR	−2.85 (−9.39–0.00)	−2.52 (−9.08–0.00)	−3.48 (−9.99–0.00)	0.14
CRP	65.03 (24.59–130.24)	63.08 (24.54–124.39)	70.41 (24.79–140.26)	0.21
CRP NA, *n* (%)	30 (1.5%)	24 (1.8%)	6 (0.9%)	
Exitus	290 (14.8%)	194 (14.8%)	96 (14.9%)	1
ICU	146 (7.5%)	91 (6.9%)	55 (8.5%)	0.25

Abbreviations: SaO_2_, oxygen saturation; NLR, neutrophil-lymphocyte ratio; NPR, neutrophil-platelets ratio; VNLR, velocity neutrophil-lymphocyte ratio; VNPR; velocity neutrophil-platelets ratio; CRP, C-reactive protein; NA, not attempted; ICU, Intensive Care Unit.

**Table 2:** Baseline Demographics, clinical and laboratory characteristics training cohort. Median value (interquartile range).

	Non-Survivors (*n* = 194)	Survivors (*n* = 1116)	*p* Value
Age	83 (75–89)	66 (54–78)	<0.0001
Sex (Male)	130 (67%)	656 (58.8%)	0.037
SaO_2_ < 90%	99 (51%)	206 (18.5%)	<0.0001
NLR	8.74 (4.65–14.96)	3.96 (2.59–6.86)	<0.0001
High (>6.9) *	117 (60.3%)	278 (24.9 %)	<0.0001
NPR	3.50 (2.41–4.93)	2.18 (1.58–3.03)	<0.0001
High (>3.0) *	116 (59.8%)	293 (26.3%)	<0.0001
VNLR	0.00 (0.00–26.61)	0.00 (−9.09–4.67)	<0.0001
*Positive (>0) #*	93 (47.9%)	365 (32.7%)	<0.0001
VNPR	0.00 (−1.70–8.78)	−3.75 (−9.70–0.00)	<0.0001
*Positive (>0) #*	75 (38.7%)	240 (21.5%)	<0.0001
CRP	114.94 (71.17–218.78)	53.80 (21.63–111.83)	<0.0001
High (>112) *	103 (53.1%)	279 (25%)	<0.0001
ICU	25 (12.9%)	66 (5.9%)	0.00074

* NLR, NPR and CRP values classified as high using the third quartile. # VNLR and VNPR categorized as positive (>0). Abbreviations: SaO_2_, oxygen saturation; NLR, neutrophil-lymphocyte ratio; NPR, neutrophil-platelets ratio; VNLR, velocity neutrophil-lymphocyte ratio; VNPR; velocity neutrophil-platelets ratio; CRP, C-reactive protein (mg/L); ICU, Intensive Care Unit.

**Table 3:** Results from the multivariate logistic regression model for the models based in NLR and NPR.

	Model NLR		Model NPR	
	OR (95% CI)	*p* Value	OR (95% CI)	*P* Value
Age	1.10 (1.09–1.12)	< 0.0001	1.11 (1.09–1.13)	<0.0001
Sex (Male)	1.87 (1.26–2.78)	0.00182	1.76 (1.19–2.63)	0.0052
SaO_2_ (<90%)	2.95 (2.00–4.34)	< 0.0001	2.83 (1.92–4.18)	<0.0001
NLR/NPR (High *)	1.90 (1.29–2.79)	0.0011	2.18 (1.49–3.17)	<0.0001
CRP (High *)	2.03 (1.37–3.02)	0.0004	2.17 (1.47–3.19)	<0.0001
	**Model VNLR**		**Model VNPR**	
	**OR (95% CI)**	***p* Value**	**OR (95% CI)**	***p* Value**
NLR/NPR score	653 (281–1576)	< 0.0001	692 (298–1672)	<0.0001
VNLR/VNPR (Positive #)	2.02 (1.40–2.92)	0.0002	2.74 (1.86–4.04)	<0.0001

* NLR, NPR and CRP values classified as high using the third quartile. # VNLR and VNPR categorized as positive (>0). Abbreviations: SaO_2_, oxygen saturation; NLR, neutrophil-lymphocyte ratio; VNLR, velocity neutrophil-lymphocyte ratio. NPR, neutrophil-platelet ratio; VNPR; velocity neutrophil-platelets ratio NLR; CRP, C-reactive protein.

## Data Availability

The data presented in this study are available on request from the corresponding author.

## Supplementary Materials

The following are available online at https://www.mdpi.com/article/10.3390/diagnostics11040596/s1, Figure S1: Diagnosis plots from imputation, Figure S2: Correlation of continuous variables with mortality, Figure S3: Sensitivity analysis results comparing the odds ratio of the model built with all the patients in the training set and only with the PCR tested patients.

## Abbreviations

Coronavirus disease 2019 (COVID-19); Intensive Care Unit (ICU); Neutrophil-to-Lymphocyte ratio (NLR); Platelet-to-lymphocyte ratio (PLR); Neutrophil-to-platelet ratio (NPR); Risk of In-hospital Mortality Score in COVID 19 (RIM Score); Velocity of NPR (VNPR); Velocity of NLR (VNLR); C-reactive protein (CRP); Polymerase Chain Reaction (PCR); Area under curve (AUC); Receiver operating characteristic curve (ROC curve)
